# Supplementary material for: Preserving exposed hydrophilic bumps on multi-bioinspired slippery surface arrays unlocks high-efficiency fog collection and photocatalytic cleaning
Source: Nat Commun. 2025 Nov 6;16:9793. doi: 10.1038/s41467-025-65169-1 (PMC12592700; doi:10.1038/s41467-025-65169-1)
Supplement: Supplementary file 1 — Supplementary Information [file 41467_2025_65169_MOESM1_ESM.pdf]

Supplementary Information

Preserving exposed hydrophilic bumps on multi-bioinspired slippery surface arrays unlocks high-efficiency fog collection and photocatalytic cleaning

Junda Wu[1], Chunxiang Li[1], Jiangdong Dai[1]*, Yan Yan[1]*

[1]Institute of Green Chemistry and Chemical Technology, School of Chemistry and Chemical Engineering, Jiangsu University, Zhenjiang, 212013, China

*Corresponding Authors:

Dr. Jiangdong Dai: daijd@ujs.edu.cn

Prof. Yan Yan : dgy5212004@163.com

This file includes:

Supplementary Figures S1-S28

Supplementary Tables S1-S8

Supplementary References

Supplementary Figures

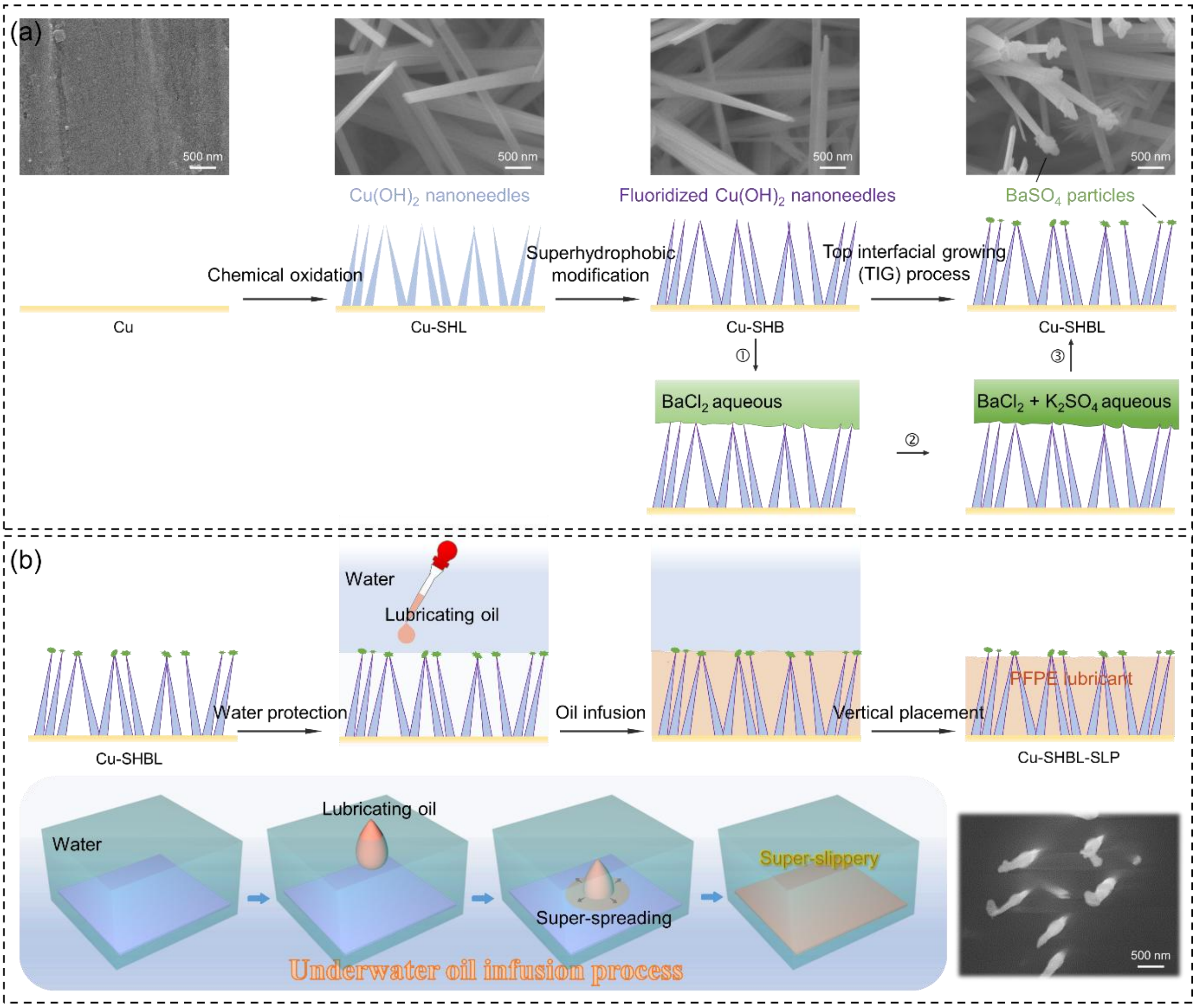

Supplementary Figure S1. Schematic illustration of the sample preparation process. (a) Preparation of Cu-SHBL film substrate. **(b)** Underwater PFPE lubricating oil infusion of Cu-SHBL film. The whole fabrication process was based on the unique surface wettability of the sample surface. Cu sheet was as the substrate and chemical oxidated and fluorinated to obtain the superhydrophobic Cu/$Cu(OH)_2$ nanorods (Cu-SHB). Take the advantage of strong water repellence of Cu-SHB, top interfacial growing (TIG) process was first proposed. The prepared $BaCl_2$ aqueous was firstly poured onto the surface of Cu-SHB film within a glass tube, enabled the aqueous could only contact the top of nanorods due to the high superhydrophobic of $Cu(OH)_2$ nanorods. Then K_2SO_4 solution was gently introduced to realize the growth of $BaSO_4$ precipitate on the top of superhydrophobic nanorods. This process was conducted within a maximum of eight cycles to obtain Cu-SHBL film with various Ba contents (characterized by the EDS analysis in Supplementary Table S1).

Subsequently, the prepared Cu-SHBL was forced underwater and conducted the PFPE lubricating oil infusion process. The hydrophilic components could attract water molecules in aqueous environment, and performed superoleophobicity underwater[1]. Therefore, during the underwater lubricant infusion process, the lubricating oil could be repelled by $BaSO_4$ hydrophilic bumps and kept spherical underwater, which provided maximal Laplace pressure (P_L) driven by the beneath nanorods (Scheme 1b). Meanwhile, compared to conventional oil infusion in air, the proposed underwater lubricating oil infusion could provide additional water hydrostatic pressure (P_H) underwater. Once the total force of P_L and P_H larger than the intrusion pressure (P_I), the lubricating oil could contact to the superoleophilic nanorods below, and rapidly permeate and spread among the nanorod arrays, which was mainly subjected to the capillary pressure (P_C) additionally, contributed to the stable and impacted SLIPS.

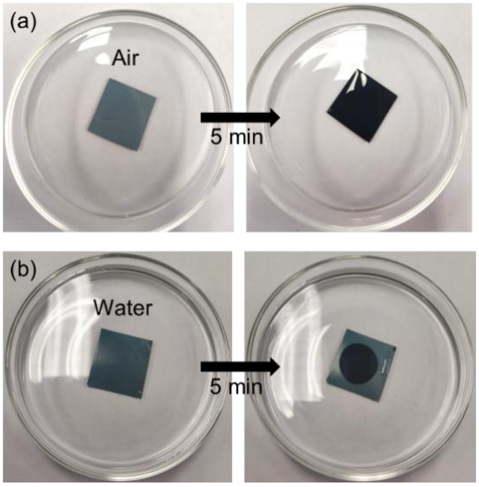

Supplementary Figure S2. **Optimal images comparation. (a)** The Cu-SHBL as the substrate and conducted the PFPE lubricating oil infusion tests in air and **(b)** in water conditions, respectively. After infusing a small and equivalent amount of PFPE lubricating oil, the oil could spread among the surface layer in air condition, while only wet and spread on the limited area in water condition. This phenomenon explained that more lubricating oil could be obtained when infusing sufficient PFPE lubricating oil in water condition compared to the air-infusion counterpart.

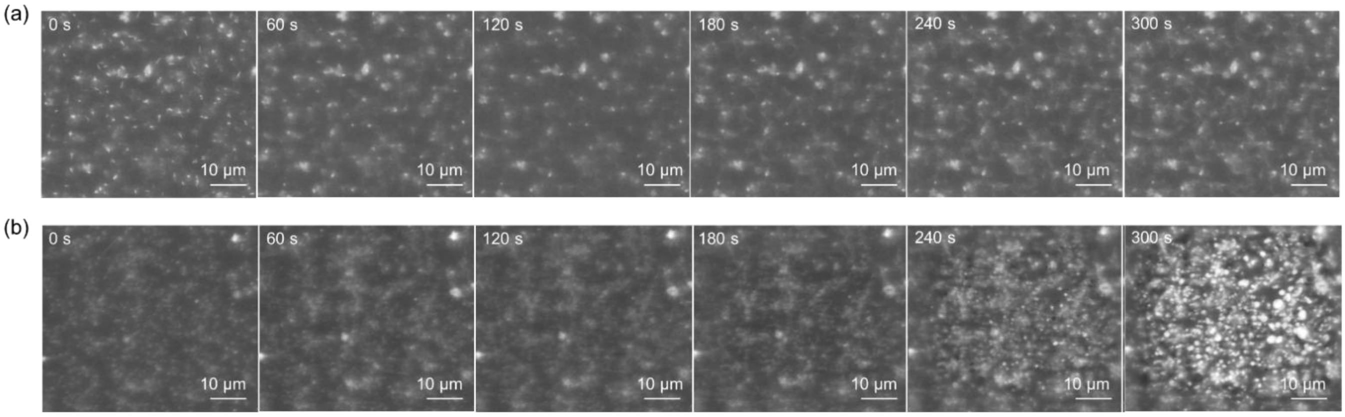

Supplementary Figure S3. Fog capture process observed by ESEM. (a) E-SEM images of Cu-SHB-SLP and **(b)** Cu-SHBL-SLP film (details of the process see Supplementary Movie S1 and 2). The temperature was reduced to –1 ± 0.5°C, and the pressure was set as 600 Pa during the ESEM tests. All the images and videos were captured at the same temperature and pressure conditions. The water droplets could be captured on the Cu-SHBL-SLP film surface at around 240 s. As a comparation, there was no obvious change on the Cu-SHB-SLP film surface, which indicated that the hydrophilic $BaSO_4$ component was liable to capture tiny water droplets in such conditions.

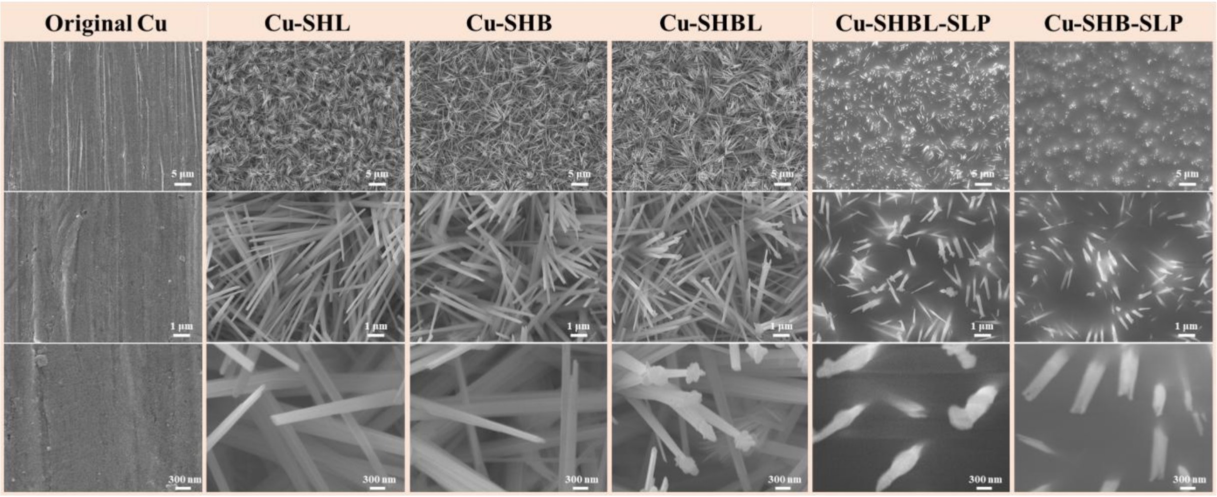

Supplementary Figure S4. Surface morphology analysis. The surface of the original Cu sheet was smooth. After the chemical etching, the $Cu(OH)_2$ nanorod arrays could be formed and named as Cu-SHL film. The PFTS superhydrophobic modification could not change the surface morphology and remained the shape of nanorods, which was named as Cu-SHB film. Through TIG process, the $BaSO_4$ nanoparticles could be formed on the top of $Cu(OH)_2$ nanorods, and named as Cu-SHBL film. After the underwater PFPE lubricating oil infusion, the interspace of nanorod arrays were filled with oil, while the hydrophilic $BaSO_4$ nanoparticles were intact and remained well on the top of nanorods, and named as Cu-SHBL-SLP. As a comparation, the Cu-SHB film without $BaSO_4$ particles was also infused lubricant underwater, and named as Cu-SHB-SLP film. The SEM image was representative of three independent analysis that yielded similar result.

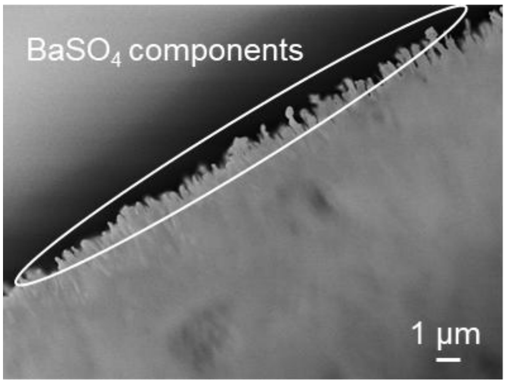

Supplementary Figure S5. Cross-sectional morphology of the Cu-SHBL film. The $BaSO_4$ particles components were successfully deposited onto the top of fluorinated $Cu(OH)_2$ nanorods via proposed TIG process. The SEM image was representative of three independent analysis that yielded similar result.

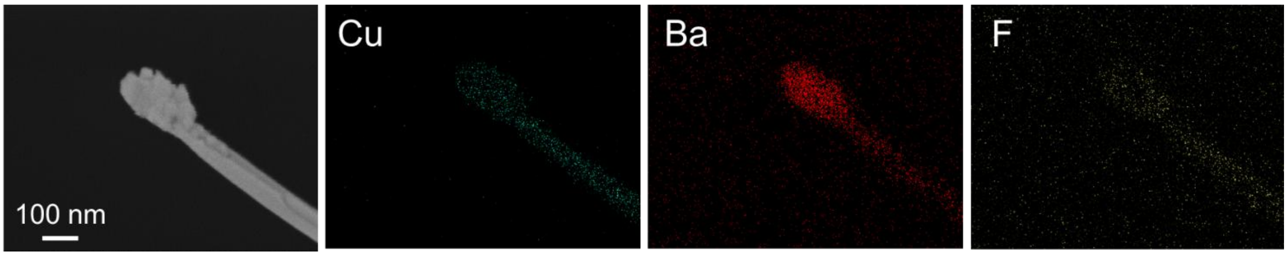

Supplementary Figure S6. EDS mapping images of Cu-SHBL film. The Ba element was concentrate on the top of fluorinated $Cu(OH)_2$ nanorod, indicated that the $BaSO_4$ component was successfully fabricated on the top via TIG process.

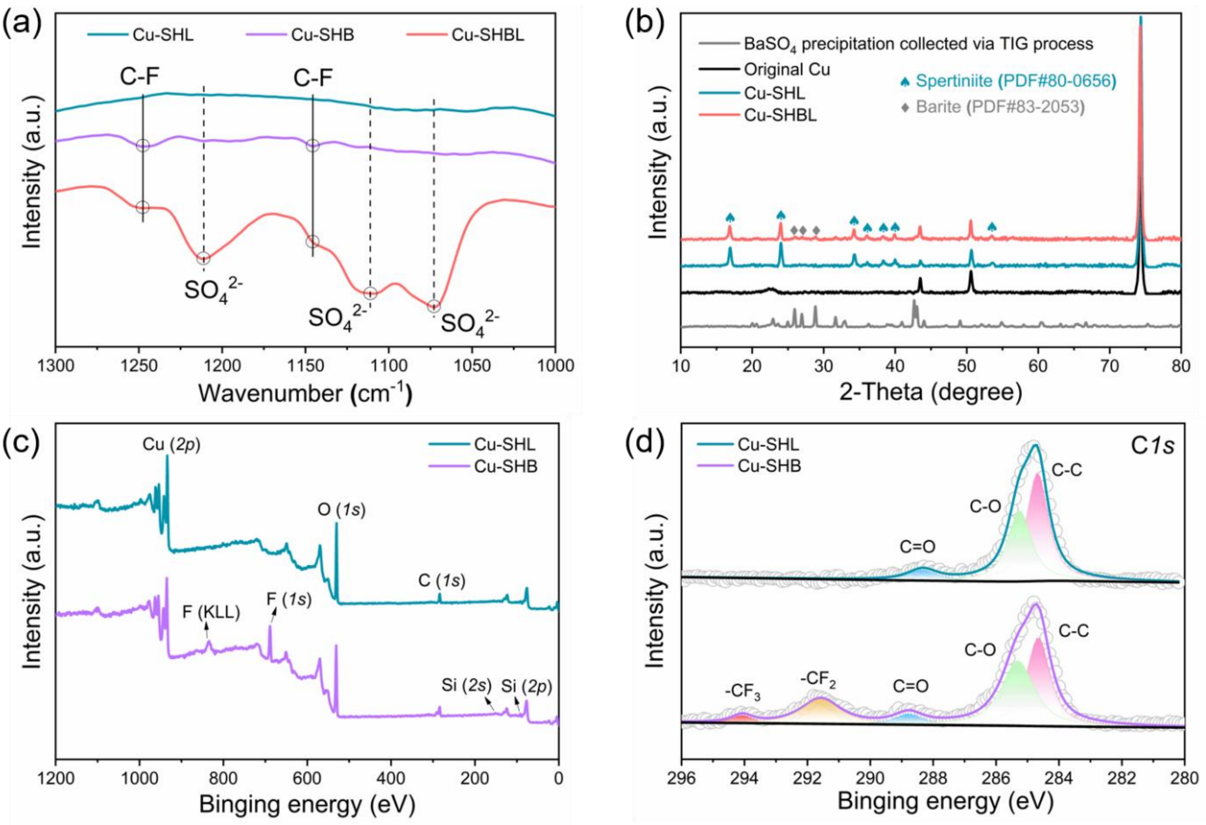

Supplementary Figure S7. Chemical components analysis. (a) FTIR spectrums of Cu-SHL, Cu-SHB and Cu-SHBL film, **(b)** XRD patterns of $BaSO_4$ deposition, original Cu, Cu-SHL and Cu-SHBL film, **(c)** XPS spectra of Cu-SHL and Cu-SHB film, **(d)** C1s spectra of Cu-SHL and Cu-SHB film. The peaks at 1247.43 and 1145.28 cm^{-1} were attributed to the stretching vibration of C-F. The peaks at 1211.44, 1109.51 and 1072.96 cm^{-1} were ascribed to the symmetrical vibrations of sulfate radical[2] (Supplementary Figure S7a). Meanwhile, the abundant characteristic peaks of hydroxyl groups in the FTIR spectrum might be the main reason of the superhydrophilic and underwater superoleophobic property of $BaSO_4$ sediment particles (Supplementary Figure S8 and S9). The C1s XPS spectra of Cu-SHL film could be deconvoluted into three peaks at 284.68, 285.25 and 288.34 eV, which was assigned to the C-C, C-O and C=O groups, respectively. On the contrast, two additional peaks at 291.58 and 294.07 eV in C1s XPS spectrum of Cu-SHB film were ascribed to the -CF_2 and -CF_3 groups, indicated that the Cu/$Cu(OH)_2$ film was successfully modified by PFTS[3]. In the X-ray diffraction (XRD) patterns (Supplementary Figure S7b), the peaks at 43.5, 50.6 and 74.3° were assigned to the characteristic peaks of the original Cu sheet. The peaks located at 16.9, 24.0, 34.3, 36.1, 38.4, 40.0 and 53.6° were ascribed to the Spertiniite (PDF#80-0656) of $Cu(OH)_2$ nanorods, indicated that the Spertiniite

crystal could be formed via chemical etching of red copper. Meanwhile, the peaks at 26.1, 27.0 and 28.6° were attributed to the Barite crystal (PDF#83-2053), confirmed the $BaSO_4$ component was successfully constructed on the $Cu(OH)_2$ nanorods top surface. These surface chemical characterization results indicated that the $BaSO_4$ deposition was successfully fabricated on the top of fluoroalkylated $Cu(OH)_2$ nanorods.

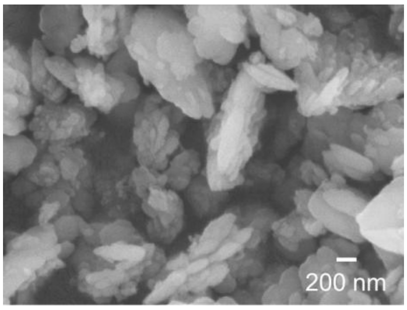

Supplementary Figure S8. Morphology of $BaSO_4$ precipitation particles. The $BaSO_4$ precipitation was collected in the solution during the TIG process, and the size was coincidence with the prepared $BaSO_4$ component particles on the nanorods top. The SEM image was representative of three independent analysis that yielded similar result.

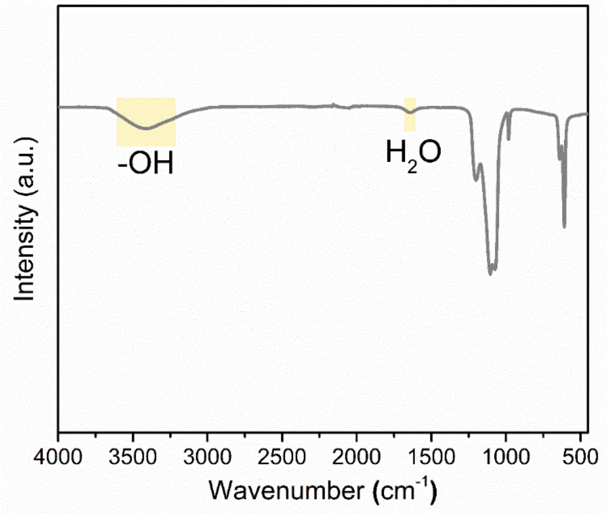

Supplementary Figure S9. FTIR spectrum of $BaSO_4$ precipitation particles. $BaSO_4$ precipitation particles was collected during the TIG process. The abundant hydroxyl group was the main reason for its hydrophilic.

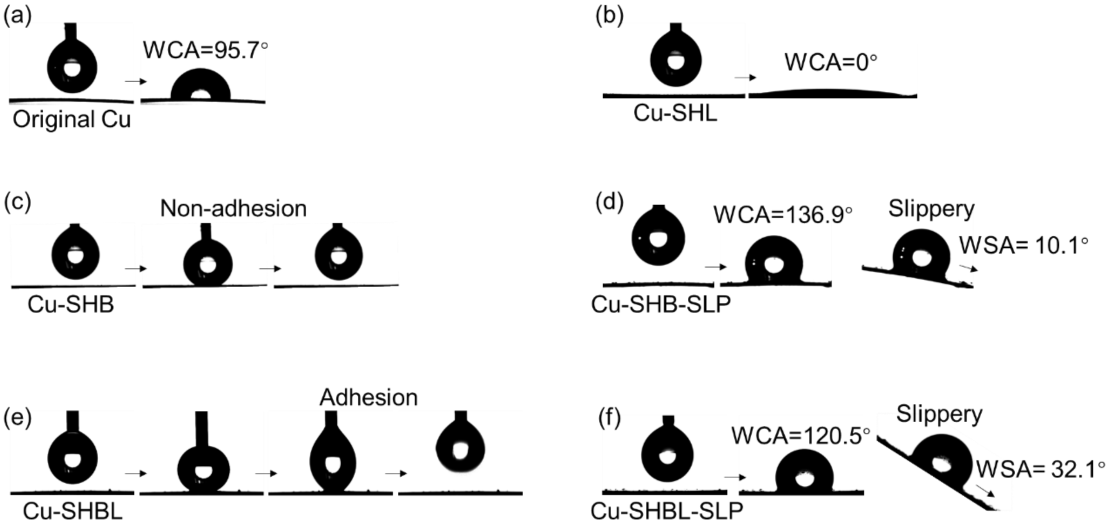

Supplementary Figure S10. Surface wettability analysis. The water contact angle (WCA) and water sliding angle (WSA) of the prepared samples: **(a)** original Cu, **(b)** Cu-SHL, **(c)** Cu-SHB, **(d)** Cu-SHB-SLP, **(e)** Cu-SHBL and **(f)** Cu-SHBL-SLP film. The original Cu sheet was hydrophobic with the WCA at 95.7°. Then the WCA changed from 95.7° to 0° owing to the high surface energy and nanosized roughness of $Cu(OH)_2$ nanorod arrays. The modification of PFTS transformed the superhydrophilic Cu-SHL to superhydrophobic Cu-SHB film while the surface remained intact, which was mainly ascribed to the molecular scale interaction between $Cu(OH)_2$ and PFTS components (Supplementary Figure S11). The water droplet even could not adhere onto the surface under external force, performed lotus-like superhydrophobic state. As a contrast, the water adhesion phenomenon on the Cu-SHBL surface was obvious under the external force, and the surface transformed to the rose petal-like superhydrophobic state. And its reflective state also could be observed underwater (Supplementary Figure S12). After the infusion of PFPE lubricating oil underwater, the WCA and WSA of Cu-SHBL-SLP were 120.5° and 32.1°, respectively. As for the Cu-SHB-SLP, there was no hydrophilic bumps on the top of nanorods, and the corresponding WCA and WSA were 136.9° and 10.1°, respectively, indicated that the design of hydrophilic bumps and SLIPS could be realized via unique underwater oil infusion process.

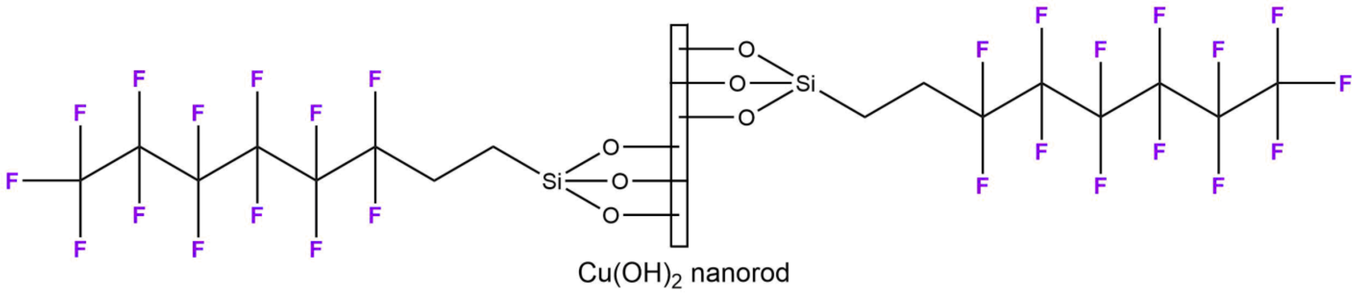

Supplementary Figure S11. Chemical molecular structure of the fluorinated $Cu(OH)_2$ nanorod. The modification of PFTS could endow the $Cu(OH)_2$ nanorods with superhydophobicity while remained the morphology intact.

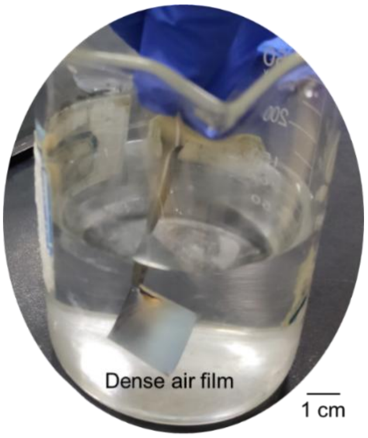

Supplementary Figure S12. Images of Cu-SHBL film forced into water condition. It could be seen that the surface of the Cu-SHBL film was reflective underwater, which was mainly ascribed to the wrapped dense air film by the superhydrophobic $Cu(OH)_2$ nanorods. The image was representative of three independent analysis that yielded similar result.

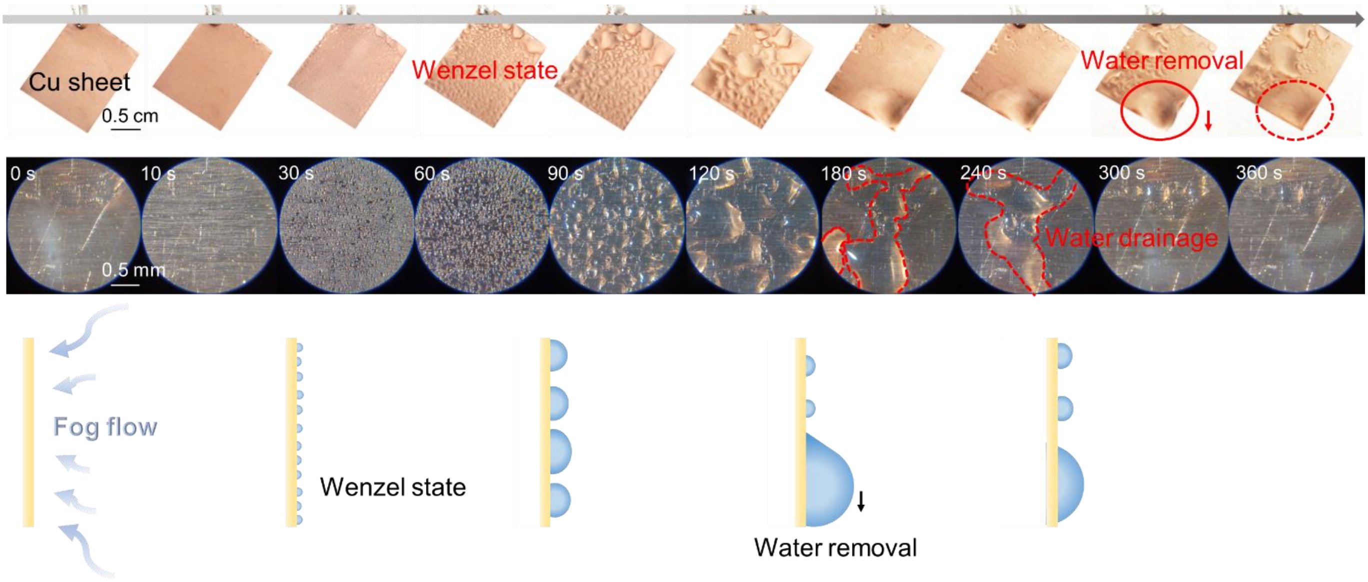

Supplementary Figure S13. Water collection process of original Cu sheet. The actual images, magnified images and process schematic diagram of Cu sheet during fog collection.

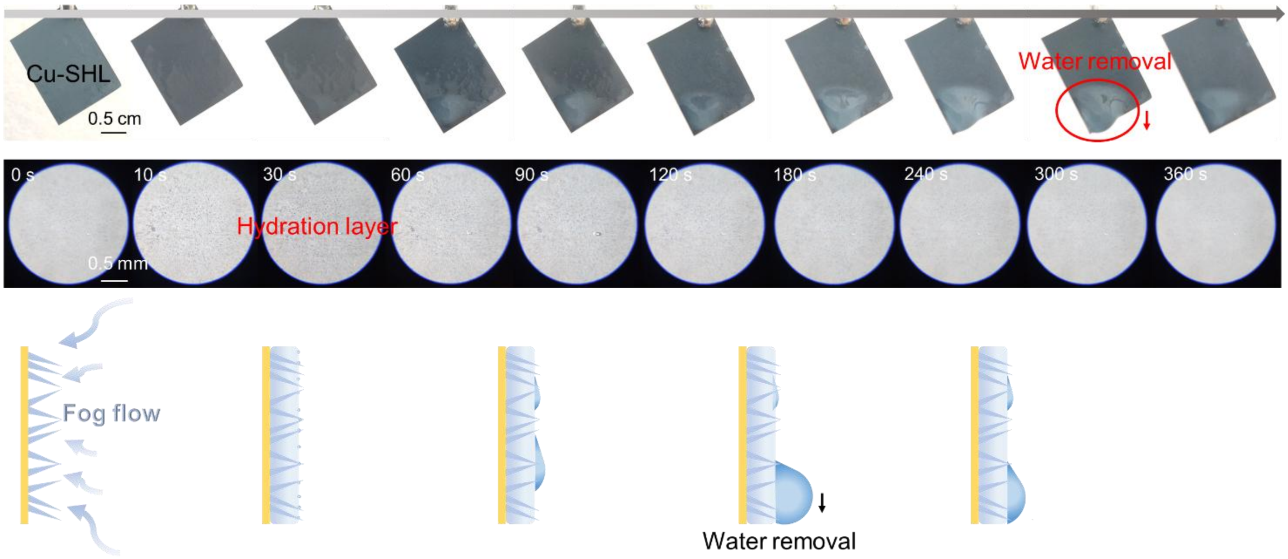

Supplementary Figure S14. Water collection process of Cu-SHL film. The actual images, magnified images and process schematic diagram of Cu-SHL film during fog collection.

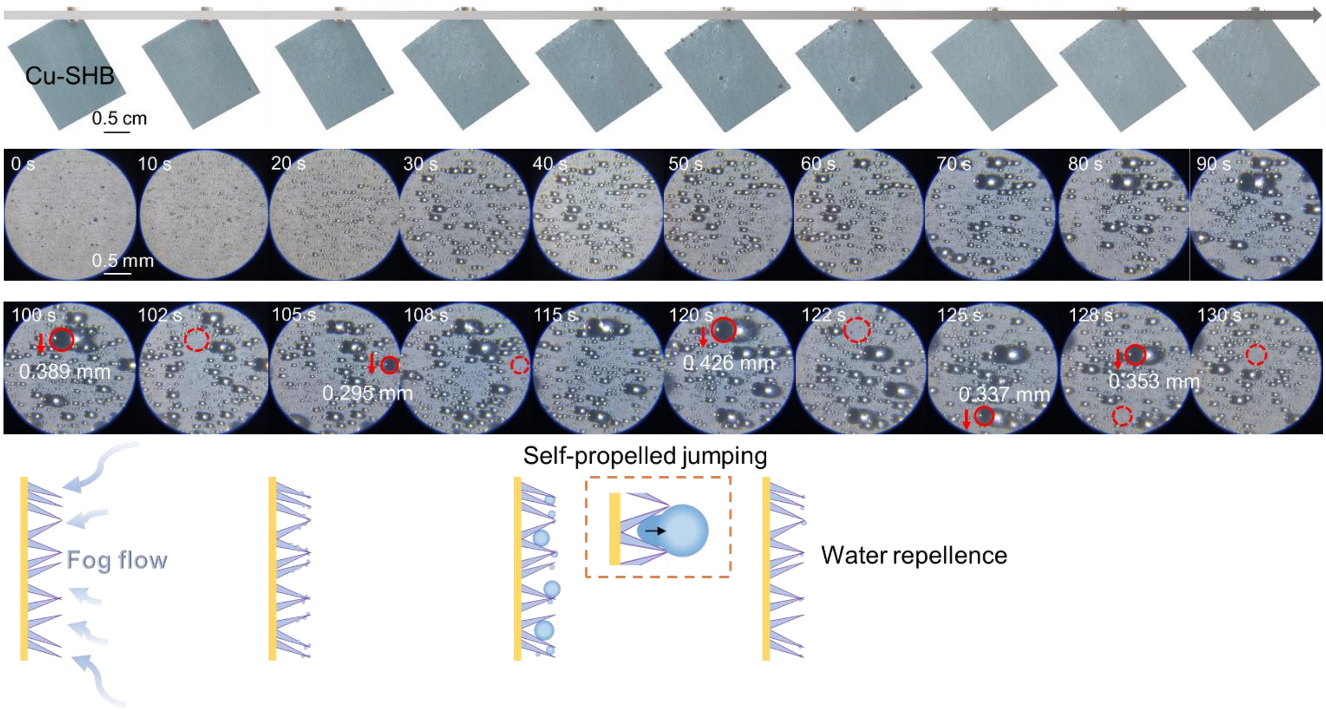

Supplementary Figure S15. Water collection process of original Cu-SHB film. The actual images, magnified images and process schematic diagram of Cu-SHB film during fog collection. The critical water droplet removal diameter could be observed in the magnified images, and were measured around 0.389, 0.295 and 0.426 mm. Due to the non-wetted property of the superhydrophobic surface, the water droplets removed from the surface could regard as spherical. And the weight could be calculated as following equation:

$$m_w = \frac{4}{3} \times \pi \times \left(\frac{d_w}{2}\right)^3 \times \rho_w \qquad (1)$$

Where the m_w (g) was the calculated droplet weight, d_w (cm) was the measured water droplet diameter, ρ_w (g/cm^{-3}) was the density of water.

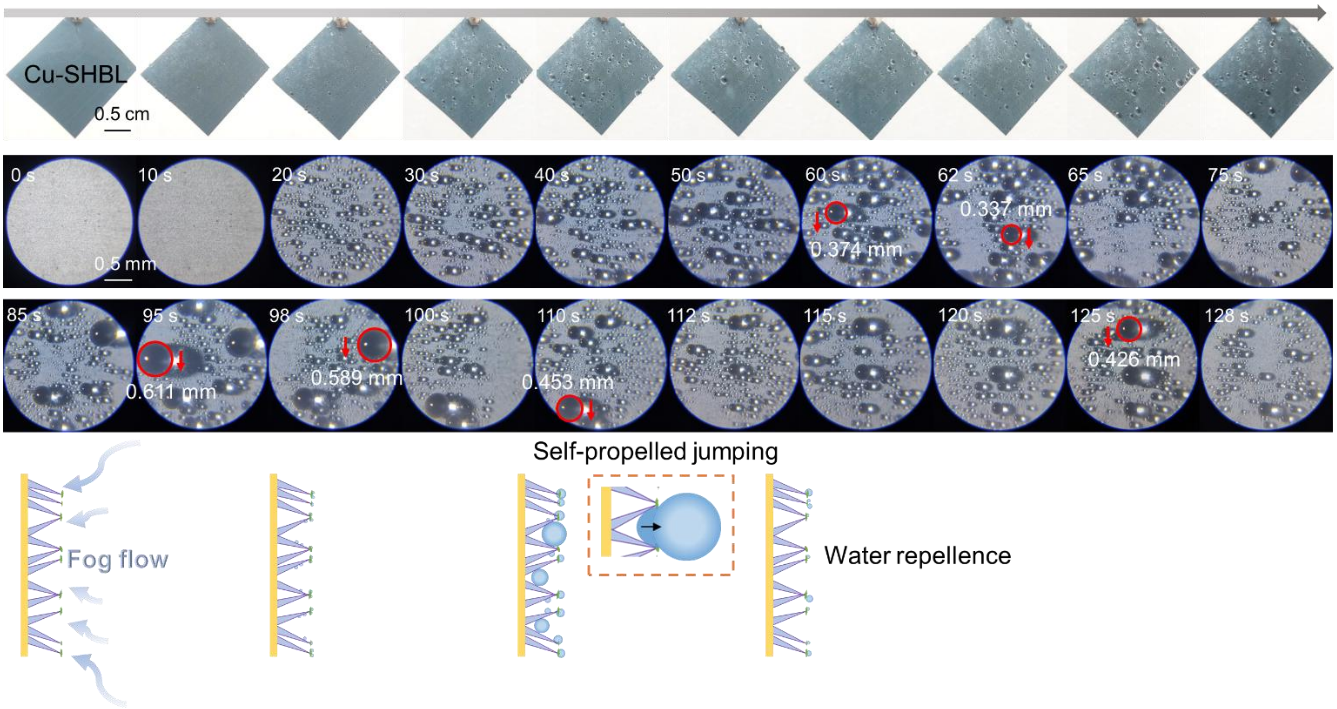

Supplementary Figure S16. Water collection process of Cu-SHBL film. The actual images, magnified images and process schematic diagram of Cu-SHBL film during fog collection. The critical water droplet removal diameter could be observed in the magnified images, and were measured around 0.374, 0.337 and 0.611 mm, and the corresponding weight could be calculated as aforementioned equation (1).

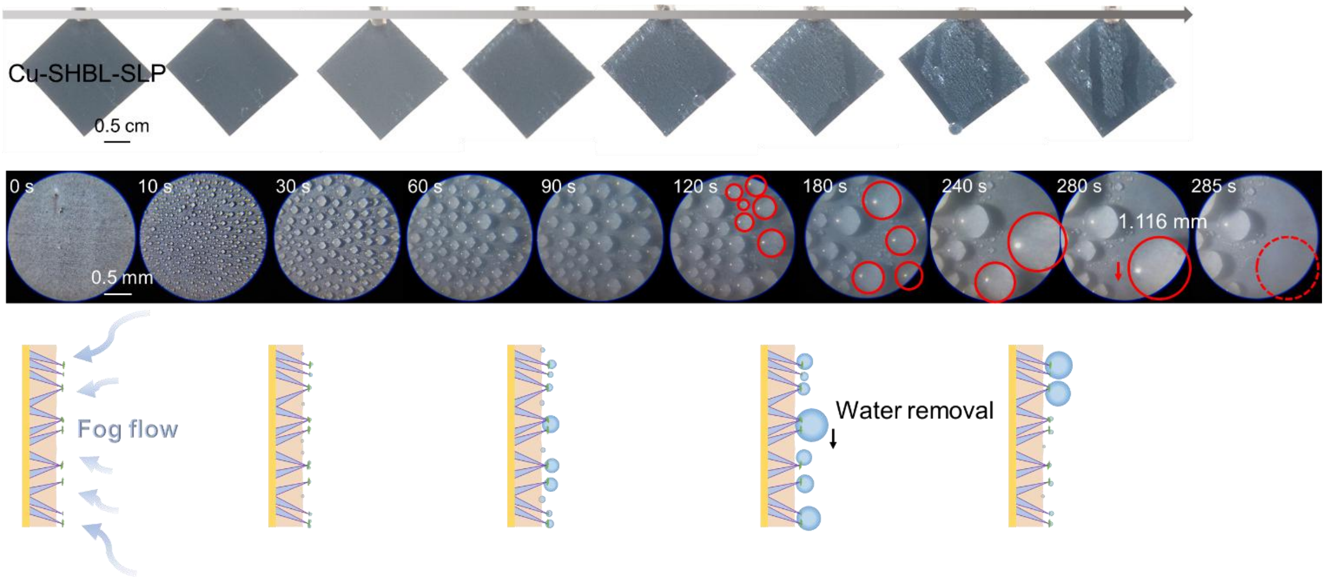

Supplementary Figure S17. Water collection process of Cu-SHBL-SLP film. The actual images, magnified images and process schematic diagram of Cu-SHBL-SLP film during fog collection.

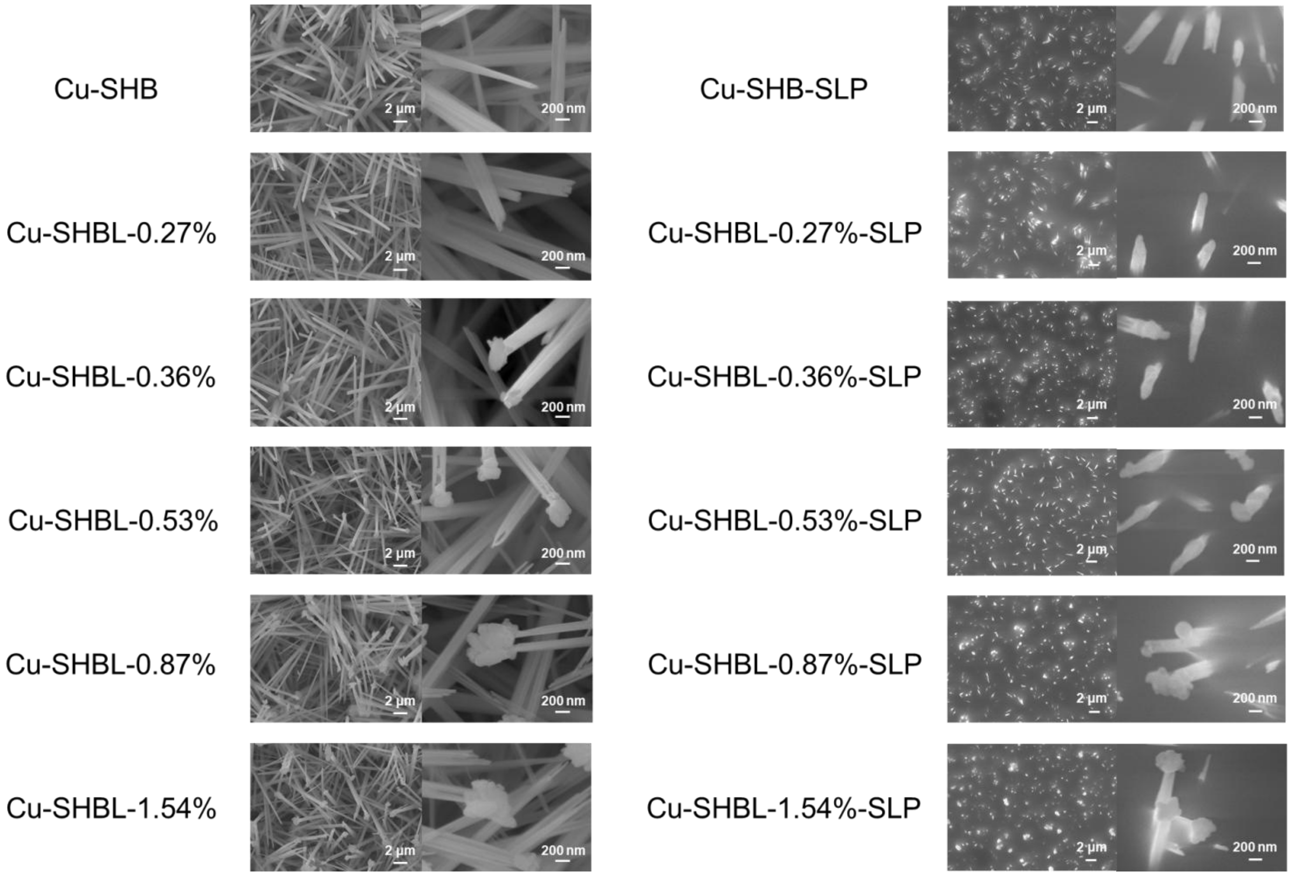

Supplementary Figure S18. Hydrophilic $BaSO_4$ bumps controllable construction. Surface morphology of Cu-SHB, Cu-SHBL-0.27%, Cu-SHBL-0.36%, Cu-SHBL-0.53%, Cu-SHBL-0.87%, Cu-SHBL-1.54% and its corresponding lubricating oil infused sheets.

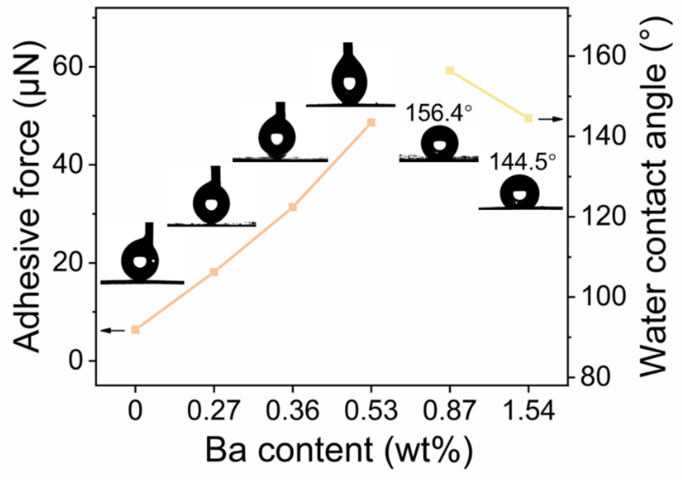

Supplementary Figure S19. Surface wettability of the samples. Water adhesive force and water contact angle of the Cu-SHBL with different Ba contents. With the Ba contents increased, the film surface become more hydrophobic.

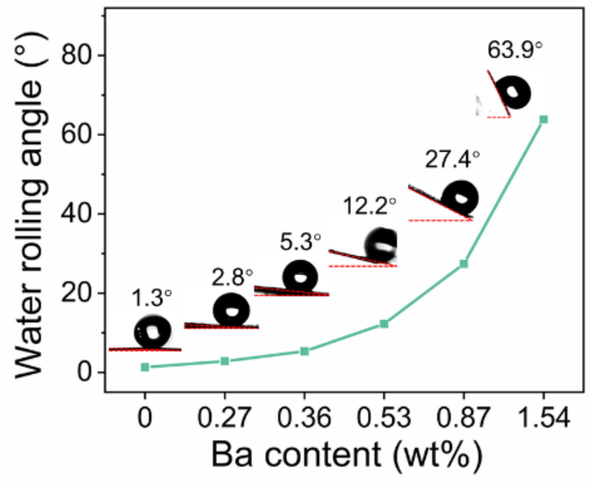

Supplementary Figure S20. Water rolling angle of the samples. With the Ba contents increased, the film surface becomes more hydrophobic.

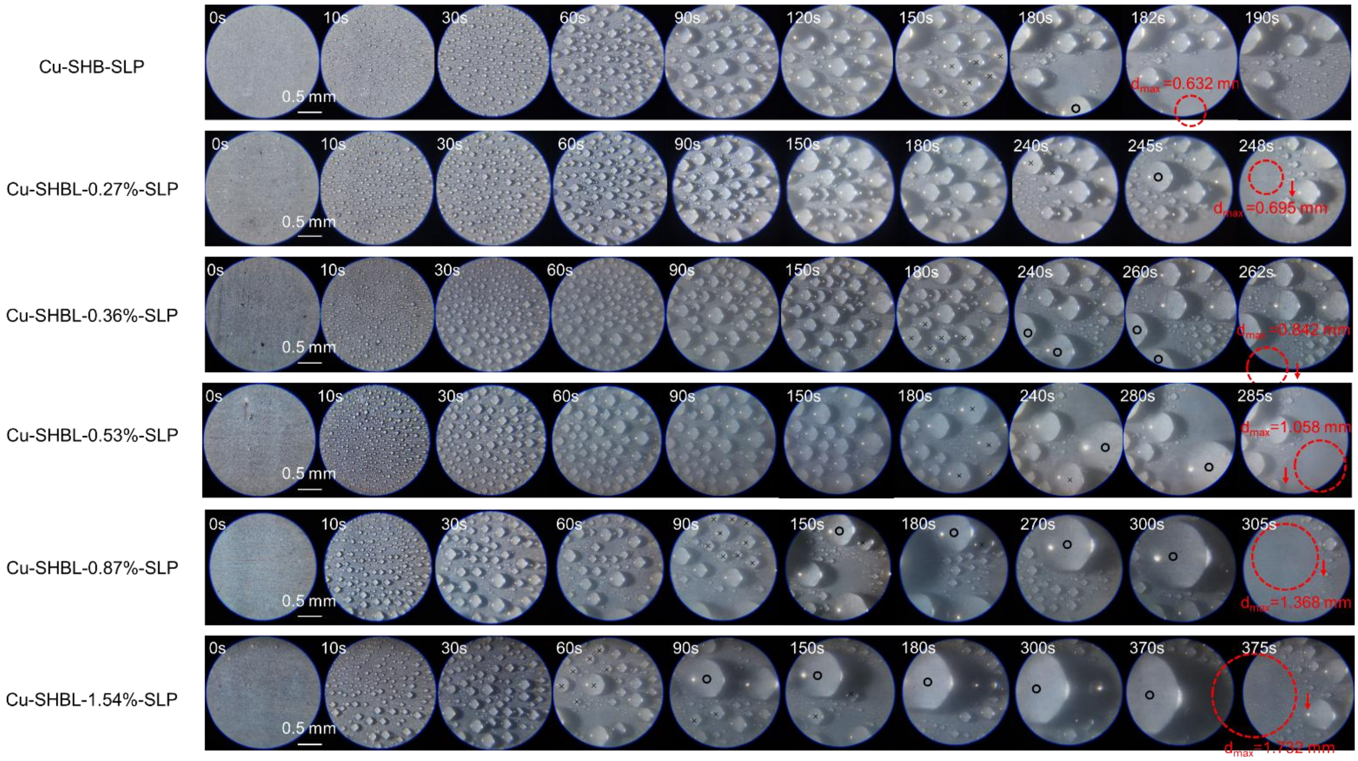

Supplementary Figure S21. **Enlarged images of water collection process.** Actual images of the fog collection process of a serious of Cu-SHBL-SLP films. The critical water droplet removal time were around 182, 248, 262, 285, 305 and 375 s, and the corresponding droplet diameter were 0.632, 0.695, 0.842, 1.058, 1.368 and 1.732 mm as for the Cu-SHB-SLP, Cu-SHBL-0.27%-SLP, Cu-SHBL-0.36%-SLP, Cu-SHBL-0.53%-SLP, Cu-SHBL-0.87%-SLP and Cu-SHBL-1.54%-SLP film, respectively.

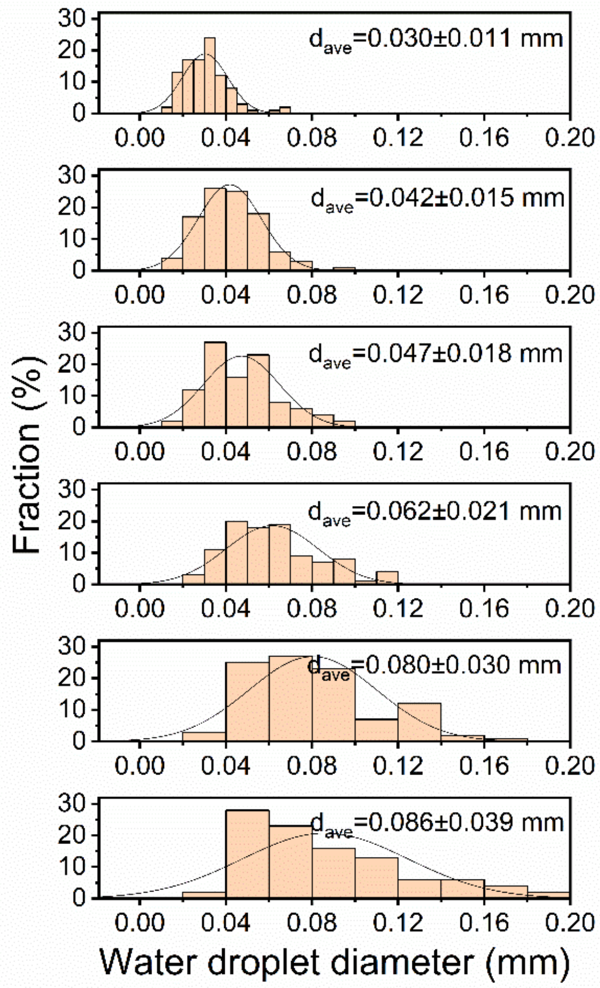

Supplementary Figure S22. Water droplets distribution analysis. Distribution of water droplets on the Cu-SHB, Cu-SHBL-0.27%-SLP, Cu-SHBL-0.36%-SLP, Cu-SHBL-0.53%-SLP, Cu-SHBL-0.87%-SLP and Cu-SHBL-1.54%-SLP film surface at 10 s during fog collection process. The average droplet increased with the hydrophilic bumps increased.

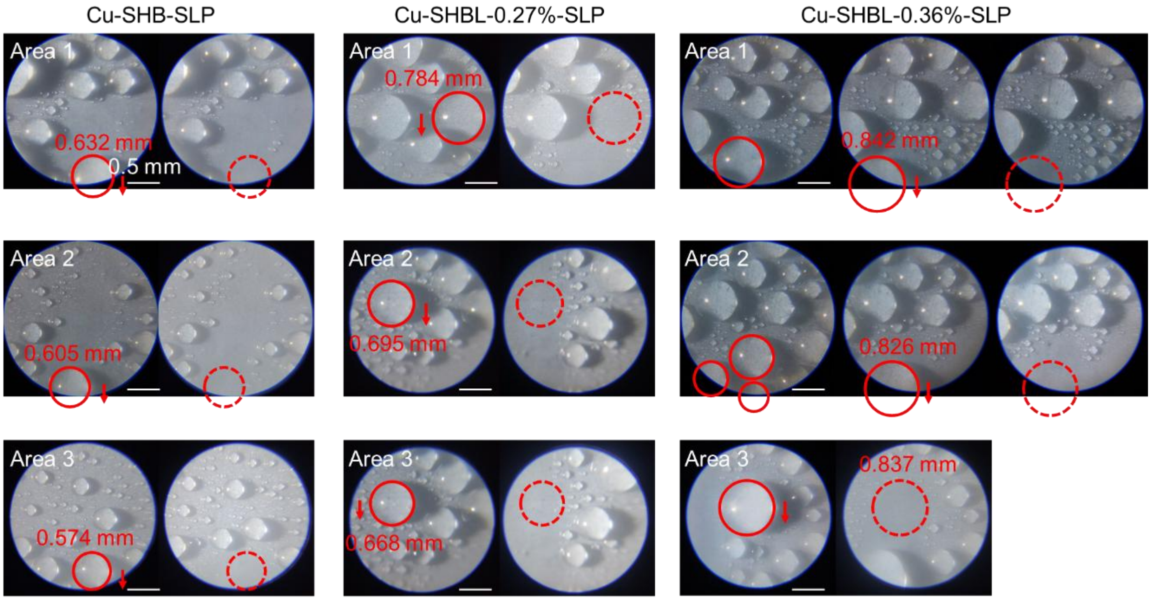

Supplementary Figure S23. Enlarged images of water collection process on different samples. Water droplet critical removal size at three random areas of Cu-SHB-SLP, Cu-SHBL-0.27%-SLP and Cu-SHBL-0.36%-SLP films. Due to the non-wetted property of the SLIPS, the water droplet was in the shape of spherical on the SLIPS, and the water adhesion force could be calculated as following equation:

$$F_h = G_w = m_w \times g = \frac{4}{3} \times \pi \times \left(\frac{d_w}{2}\right)^3 \times \rho_w \qquad (2)$$

Where F_h (N) was the adhesion force of the water droplet on SLIPS, d_w (m) was the measured water droplet diameter, ρ_w (g/cm^{-3}) was the density of water.

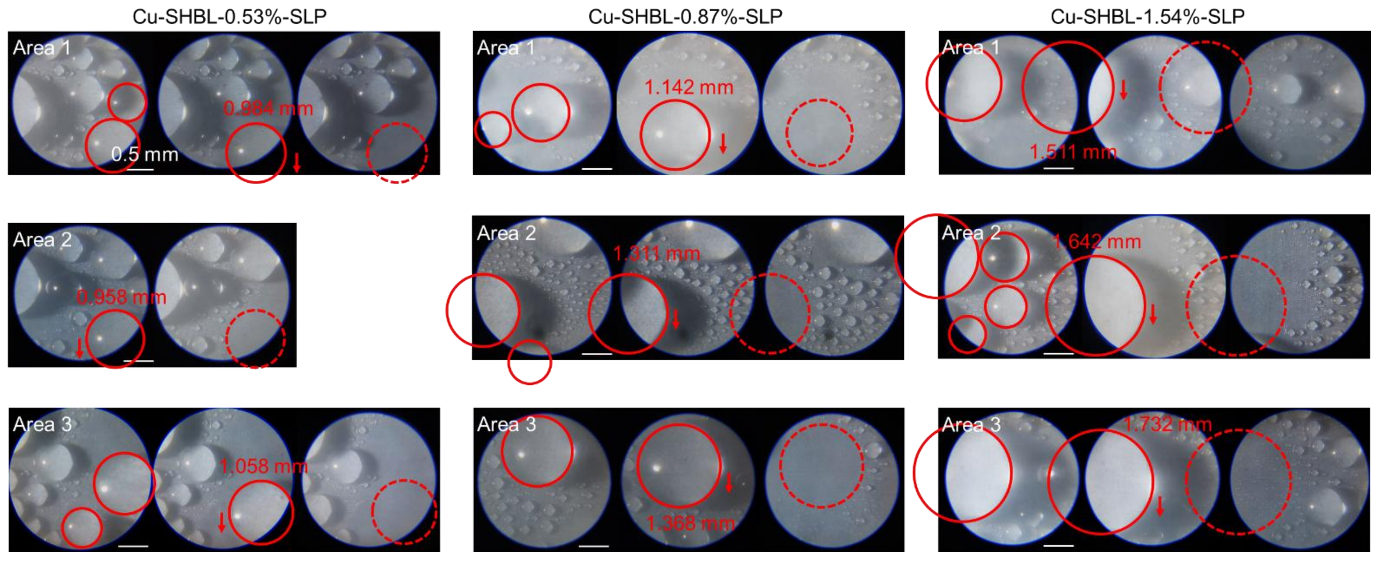

Supplementary Figure S24. **Enlarged images of water collection process on different samples.** Water droplet critical removal size at three random areas of Cu-SHBL-0.53%-SLP, Cu-SHBL-0.87%-SLP and Cu-SHBL-1.54%-SLP films. The water droplet adhesion force could be calculated as equation (2) according to the water droplet critical removal size.

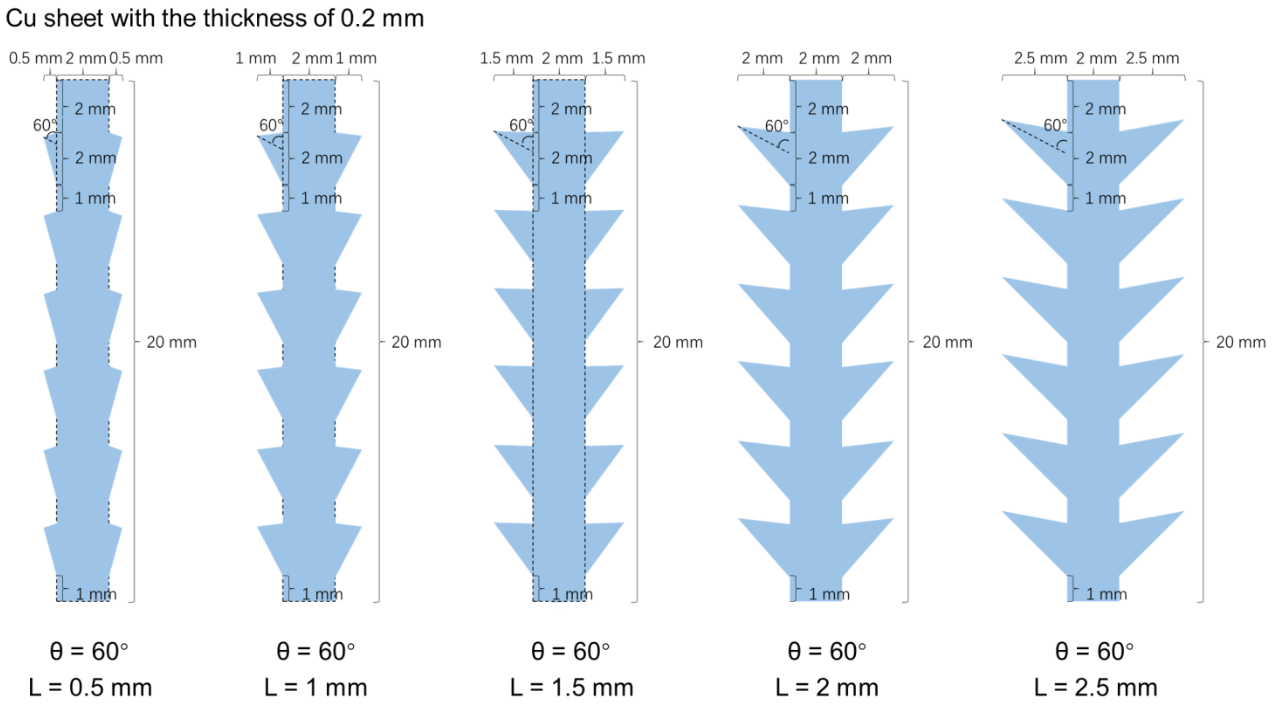

Supplementary Figure S25. Detail parameters of patterned fog collectors for laser ablation. Design of patterned fog collector with the uniform θ values and different L values.

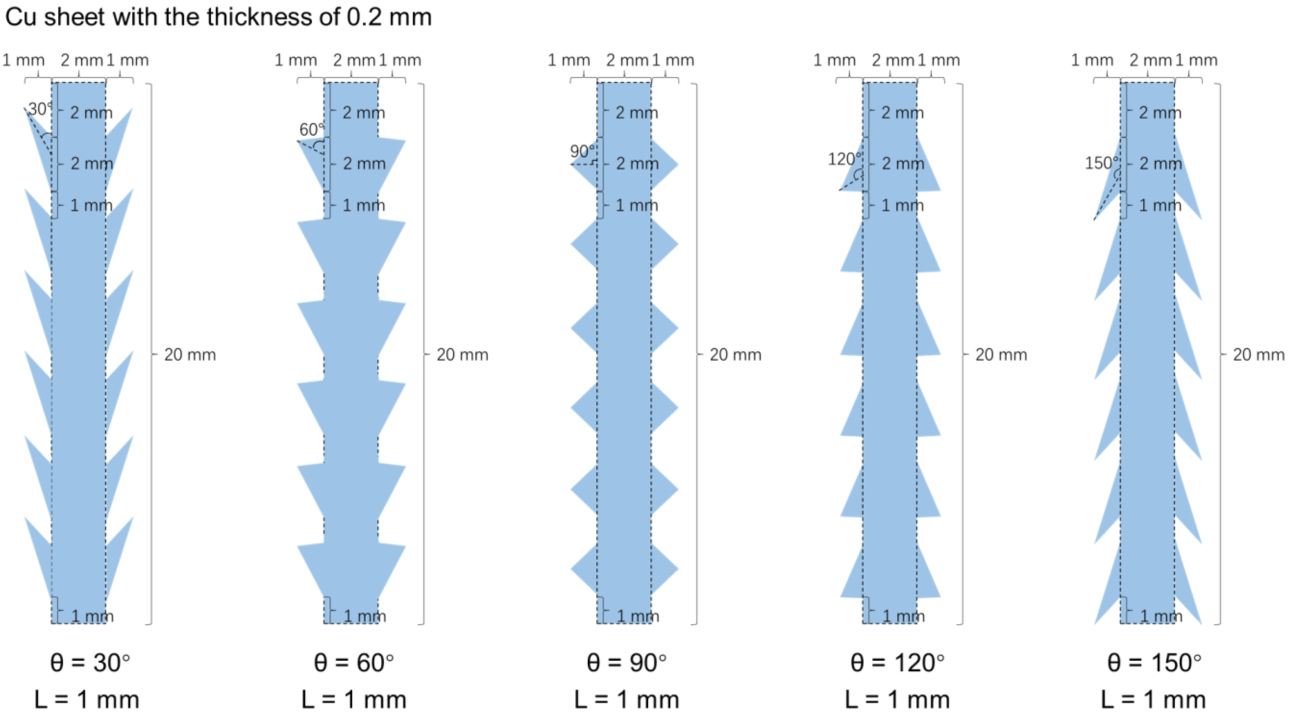

Supplementary Figure S26. Detail parameters of patterned fog collectors for laser ablation. Design of patterned fog collector with the uniform θ values and different L values.

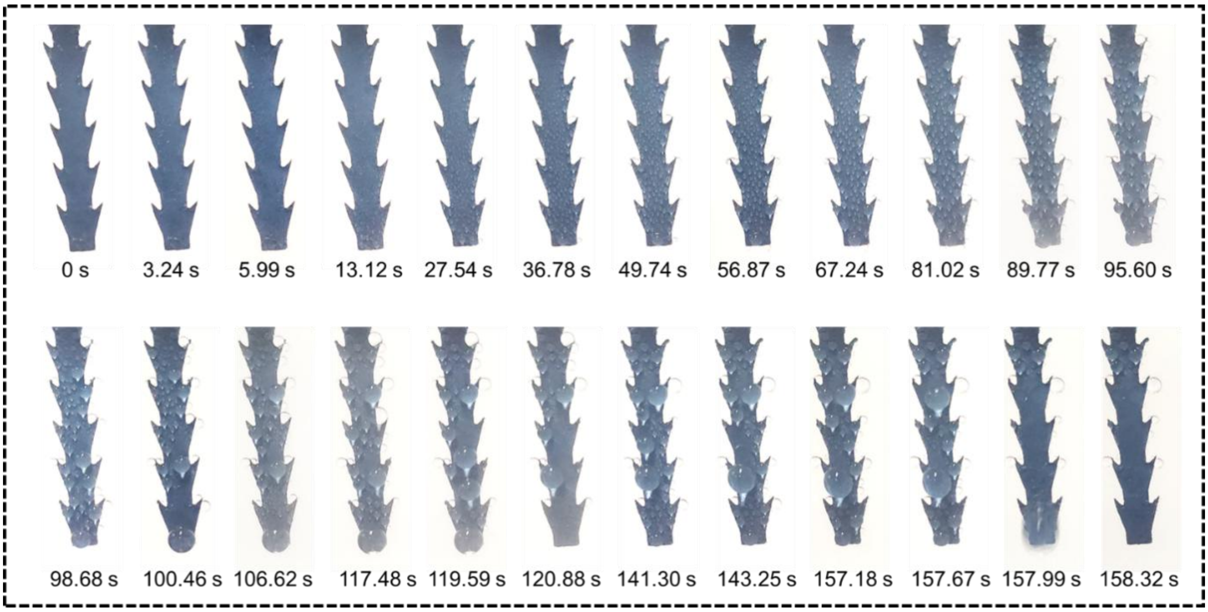

Supplementary Figure S27. Observation of the water collection process. Actual water collection process of patterned Cu-SHBL-SLP with L=1 and θ=30°.

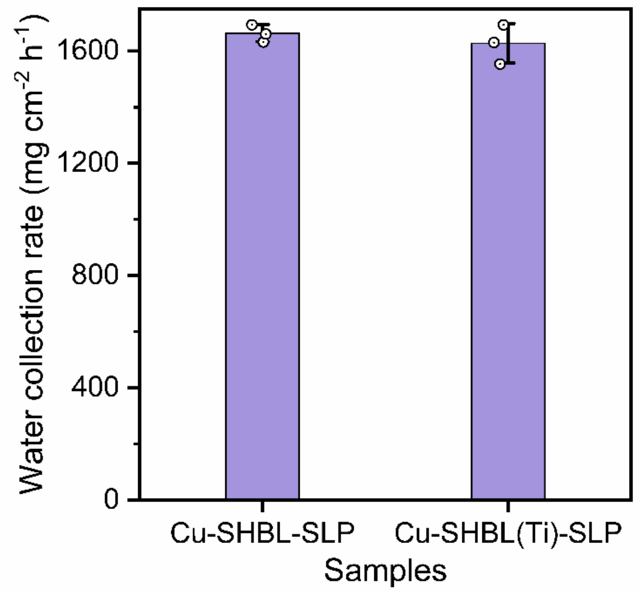

Supplementary Figure S28. Water collection rate comparation. There was no significant decline of the WCR after introducing TiO_2 particles on Cu-SHBL-SLP film. Error bars in SD (standard deviation) were obtained by statistically repeating independent identical experimental and results three times, and data were presented as mean ± SD.

Supplementary Tables

Supplementary Table S1. Summary of water collection performance of different samples. Theoretical water collection rate (mg cm^{-2} h^{-1}), experimental water collection rate (mg cm^{-2} h^{-1}) and water collection efficiency (%) of original Cu, Cu-SHL, Cu-SHB, Cu-SHBL and Cu-SHBL-SLP film.

Sample	Theoretical water collection rate (mg cm^{-2} h^{-1})	Experimental water collection rate (mg cm^{-2} h^{-1})	Water collection efficiency (%)
Cu	10610	897	8.45
Cu-SHL	10610	844	7.95
Cu-SHB	10610	1188	11.20
Cu-SHBL	10610	1318	12.42
Cu-SHBL-SLP	10610	1664	15.68

Supplementary Table S2. EDS analysis of the samples. Surface elements distribution of the prepared a serious of Cu-SHBL films measured by EDS.

Samples	Cu	O	Ba	F	Si
1	79.47	20.14	0.27	0	0.13
2	80.24	19.18	0.36	0	0.23
3	84.88	14.36	0.53	0.01	0.22
4	78.12	20.62	0.87	0.01	0.38
5	77.58	20.25	1.54	0.11	0.53

Supplementary Table S3. Summary of water collection performance of different samples. Theoretical water collection rate (mg cm^{-2} h^{-1}), experimental water collection rate (mg cm^{-2} h^{-1}) and water collection efficiency (%) of Cu-SHB-SLP, Cu-SHBL-0.27%-SLP, Cu-SHBL-0.36%-SLP, Cu-SHBL-0.53%-SLP, Cu-SHBL-0.87%-SLP and Cu-SHBL-1.54%-SLP film.

Sample	Theoretical water collection rate (mg cm^{-2} h^{-1})	Experimental water collection rate (mg cm^{-2} h^{-1})	Water collection efficiency (%)
Cu-SHB-SLP	10610	1250	11.78
Cu-SHBL-0.27%-SLP	10610	1306	12.31
Cu-SHBL-0.36%-SLP	10610	1510	14.23
Cu-SHBL-0.53%-SLP	10610	1664	15.68
Cu-SHBL-0.87%-SLP	10610	1508	14.21
Cu-SHBL-1.54%-SLP	10610	1382	13.02

Supplementary Table S4. Summary of water collection performance of different samples. Theoretical water collection rate (mg cm^{-2} h^{-1}), experimental water collection rate (mg cm^{-2} h^{-1}) and water collection efficiency (%) of 2D patterned Cu-SHBL-SLP film with different L values.

Sample	Theoretical water collection rate (mg cm^{-2} h^{-1})	Experimental water collection rate (mg cm^{-2} h^{-1})	Water collection efficiency (%)
Cu-SHBL-SLP (L=0.5 cm, θ=60°)	10610	4546	42.85
Cu-SHBL-SLP (L=1.0 cm, θ=60°)	10610	5081	47.89
Cu-SHBL-SLP (L=1.5 cm, θ=60°)	10610	4561	42.89
Cu-SHBL-SLP (L=2.0 cm, θ=60°)	10610	4146	39.07
Cu-SHBL-SLP (L=2.5 cm, θ=60°)	10610	3696	34.83

Supplementary Table S5. Summary of water collection performance of different samples. Theoretical water collection rate (mg cm^{-2} h^{-1}), experimental water collection rate (mg cm^{-2} h^{-1}) and water collection efficiency (%) of 2D patterned Cu-SHBL-SLP film with different θ values.

Sample	Theoretical water collection rate (mg cm^{-2} h^{-1})	Experimental water collection rate (mg cm^{-2} h^{-1})	Water collection efficiency (%)
Cu-SHBL-SLP (L=1.0 cm, θ=30°)	10610	4941	46.57
Cu-SHBL-SLP (L=1.0 cm, θ=60°)	10610	5081	47.89
Cu-SHBL-SLP (L=1.0 cm, θ=90°)	10610	4461	42.04
Cu-SHBL-SLP (L=1.0 cm, θ=120°)	10610	4129	38.92
Cu-SHBL-SLP (L=1.0 cm, θ=150°)	10610	3355	31.62

Supplementary Table S6. Working comparation of fog collector without external energy input.

Label	Material categories	Name	Fog flow (mL/h)	WCR (mg m^{-2} h^{-1})	Ref.
1	3D structure	MN-HB/HL	40	3050	4
2		CP-HL-SLIPS	100	4400	5
7		Conical CS-EVA	225	10400	6
10		SLP-SHL@SP-Cu	300	7421	7
15		Bioinspired cactus kirigami	400	4000	8
3	2D membrane	CM@PDMS-PS	200	907	9
4		PP@pTA/SNFs/PFDT	230	2130	10
6		Cu_2O/ZrO_2 hybrid surface	252	1707	11
11		AWF-6	350	1538	12
16		FSLC	1080	1930	13
19		A-HH-LIS	1800	9600	14
5	Janus structure	WCJM	252	1100	15
9		S-Z-STA	300	2479	16
17		PFC	1080	4210	17
18		J-LV devices	1200	1230	18
8	Harps	PNIPAm-PVDF harps	300	1415	19
12		Janus-PAN harp	350	1775	20
13		PAN-TO harp	350	4035	21
14		$PVDF-TiO_2$ yarns	400	400	22
	Our work	Cu-SHBL-SLP	300	5081	
			420	11238	
			900	25207	
			1500	61588	

Supplementary Table S7. Summary of the reported water collection performance. Fog flux (mL/h), theoretical water collection rate (mg cm^{-2} h^{-1}), experimental water collection rate (mg cm^{-2} h^{-1}) and water collection efficiency (%) of reported works.

Sample	Fog flow (mL/h)	Theoretical water collection rate (mg cm^{-2} h^{-1})	Experimental water collection rate (mg cm^{-2} h^{-1})	Water collection efficiency (%)	Refs
Long plasma-textured PMMA	25.2	4700	1171	25	[23]
PFC	1080	11800	4210	35.68	[17]
Cu-SHBL-SLP	300	10610	5081	47.89	Our work
Cu-SHBL-SLP	420	33423	11238	33.63	Our work
Cu-SHBL-SLP	900	71620	25207	35.20	Our work
Cu-SHBL-SLP	1500	119366	61588	51.60	Our work

Supplementary Table S8. Summary of the chemical reagents and materials cost for the fabrication of Cu-SHBL-SLP films.

Materials	Prices	Amount	Cost
Red copper sheet	\$ 12.1/m^2	1 m^2	\$ 12.1
NaOH	\$ 3.4/500g	0.680 kg	\$ 4.6
$(NH_4)_2S_2O_8$	\$ 4.2/500g	0.204 kg	\$ 1.7
PFTS	\$ 12.1/25g	0.011 kg	\$ 5.3
$BaCl_2$	\$ 5.6/500g	0.710 kg	\$ 7.9
K_2SO_4	\$ 4.6/500g	0.520 kg	\$ 4.7
PFPE	\$ 42.0/25g	0.024 kg	\$ 40.3
Total	-	-	\$ 76.6

The total fabrication cost for the entire process of the 2D-patterned Cu-SHBL-SLP collector has been carefully calculated**.** A single 2D-patterned Cu-SHBL-SLP collector (active area ≈ 0.646 cm^2) costs approximately 3.88 yuan (\$ 0.54) and achieves a water collection rate of 61588 mg cm^{-2} h^{-1} (fog flow at 1500 mL/h). Consequently, this corresponds to a remarkably low water production cost of ≈0.006 yuan (\$ 0.0008) per liter per square meter per hour (fog flow at 1500 mL/h), demonstrating excellent cost efficiency and scalability. For large-scale applications, the total fabrication cost of the 2D-patterned Cu-SHBL-SLP is \$ 76.6 per 1 m^2. To construct stable SLIPS, we employed PFPE lubricant, which is relatively expensive. Developing low-cost stable lubricants represents a promising research direction for practical fabrication and application, contributing to the development of highly efficient bio-inspired fog collectors. It is important to note that our current work presents a proof-of-concept demonstrating the multi-bioinspired fog collection approach; subsequent efforts focused on cost reduction and efficiency improvement are warranted.

Supplementary References

1 Su, B., Tian, Y. & Jiang, L. Bioinspired Interfaces with Superwettability: From Materials to Chemistry. *J Am Chem Soc* **138**, 1727-1748, doi:10.1021/jacs.5b12728 (2016).

2 Hu, D. *et al.* Multifunctional UV-shielding nanocellulose films modified with halloysite nanotubes-zinc oxide nanohybrid. *Cellulose* **27**, 401-413, doi:10.1007/s10570-019-02796-0 (2019).

3 Liu, Y. Q. *et al.* Laser-structured Janus wire mesh for efficient oil-water separation. *Nanoscale* **9**, 17933-17938, doi:10.1039/c7nr06110b (2017).

4 Zhou, W. *et al.* High‐Performance Freshwater Harvesting System by Coupling Solar Desalination and Fog Collection with Hierarchical Porous Microneedle Arrays. *Advanced Functional Materials* **32**, doi:10.1002/adfm.202113264 (2022).

5 Maji, K., Das, A., Dhar, M. & Manna, U. Synergistic chemical patterns on a hydrophilic slippery liquid infused porous surface (SLIPS) for water harvesting applications. *J. Mater. Chem. A* **8**, 25040-25046, doi:10.1039/d0ta09271a (2020).

6 Deng, K. *et al.* A conical shaped self-healing slippery film for enhanced fog harvesting in windy environment. *Chemical Engineering Science* **292**, doi:10.1016/j.ces.2024.119980 (2024).

7 Yu, Z. *et al.* Bio‐inspired Copper Kirigami Motifs Leading to a 2D–3D Switchable Structure for Programmable Fog Harvesting and Water Retention. *Advanced Functional Materials* **33**, doi:10.1002/adfm.202210730 (2022).

8 Bai, H. *et al.* Cactus kirigami for efficient fog harvesting: simplifying a 3D cactus into 2D paper art. *J. Mater. Chem. A* **8**, 13452-13458, doi:10.1039/d0ta01204a (2020).

9 Zhang, Y. *et al.* Lizard-Skin-Inspired Nanofibrous Capillary Network Combined with a Slippery Surface for Efficient Fog Collection. *ACS Appl Mater Interfaces* **13**, 36587-36594, doi:10.1021/acsami.1c10067 (2021).

10 Zhang, J., Li, B., Zhou, Z. & Zhang, J. Durable Superhydrophobic Surfaces with Self‐Generated Wenzel Sites for Efficient Fog Collection. *Small* **20**, doi:10.1002/smll.202312112 (2024).

11 Feng, J., Zhong, L. & Guo, Z. Sprayed hieratical biomimetic superhydrophilic-superhydrophobic surface for efficient fog harvesting. *Chemical Engineering Journal* **388**, doi:10.1016/j.cej.2020.124283 (2020).

12 Ji, Y. *et al.* Thermodynamically Induced Interfacial Condensation for Efficient Fog Harvesting. *Small* **19**, doi:10.1002/smll.202304037 (2023).

13 Ma, J., Zhang, C., Zhang, P. & Song, J. One-step synthesis of functional slippery lubricated coating with substrate independence, anti-fouling property, fog collection, corrosion resistance, and icephobicity. *Journal of Colloid and Interface Science* **664**, 228-237, doi:10.1016/j.jcis.2024.03.027 (2024).

14 Gou, X. & Guo, Z. Reed leaf-inspired anisotropic slippery lubricant-infused surface for water collection and bubble transportation. *Chemical Engineering Journal* **411**, doi:10.1016/j.cej.2021.128495 (2021).

15 Tang, X., Zhang, F., Huang, J. & Guo, Z. Multifuctional Janus Materials for Rapid One-Way Water Transportation and Fog Collection. *Langmuir* **37**, 13778-13786, doi:10.1021/acs.langmuir.1c02625 (2021).

16 Wu, J., Yan, Z., Yan, Y., Li, C. & Dai, J. Beetle-Inspired Dual-Directional Janus Pumps with Interfacial Asymmetric Wettability for Enhancing Fog Harvesting. *ACS Applied Materials & Interfaces* **14**, 49338-49351, doi:10.1021/acsami.2c14808 (2022).

17 Yan, D., Chen, Y., Liu, J. & Song, J. Super‐Fast Fog Collector Based on Self‐Driven Jet of Mini Fog Droplets. Small 19, doi:10.1002/smll.202301745 (2023).

18 Zhou, Y., Fu, S., Ai, S., Pei, K. & Guo, Z. In-situ dual-MOF functionalized quasi-fractal networks with Janus wettability for high-efficient fog harvesting and intelligent interface materials. *Chemical Engineering Journal* **498**, doi:10.1016/j.cej.2024.155489 (2024).

19 Parisi, G., Szewczyk, P. K., Narayan, S. & Stachewicz, U. Thermoresponsive nanofiber yarns for water harvesting enhanced by harp system. *Chemical Engineering Journal* **499**, doi:10.1016/j.cej.2024.155874 (2024).

20 Ji, Y. *et al.* A Molecular Confine-Induced Charged Fiber for Fog Harvesting. *Advanced Fiber Materials*, doi:10.1007/s42765-024-00474-w (2024).

21 Ji, Y. *et al.* Nanoscale Drag Reduction Effect Enables Efficient Fog Harvesting. *Advanced Functional Materials*, doi:10.1002/adfm.202411083 (2024).

22 Parisi, G., Szewczyk, P. K., Narayan, S. & Stachewicz, U. Wettability gradient of photoresponsive electrospun yarns for harp-based fog water harvesting. *Cell Reports Physical Science* **5**, doi:10.1016/j.xcrp.2024.102176 (2024).

23 Nioras, D., Ellinas, K., Constantoudis, V. & Gogolides, E. How Different Are Fog Collection and Dew Water Harvesting on Surfaces with Different Wetting Behaviors? ACS Appl Mater Interfaces 13, 48322-48332, doi:10.1021/acsami.1c16609 (2021).
