# Supplementary material for: Preserving exposed hydrophilic bumps on multi-bioinspired slippery surface arrays unlocks high-efficiency fog collection and photocatalytic cleaning
Source: Nat Commun. 2025 Nov 6;16:9793. doi: 10.1038/s41467-025-65169-1 (PMC12592700; doi:10.1038/s41467-025-65169-1)
Supplement: Supplementary file 2 — Description of Additional Supplementary File [file 41467_2025_65169_MOESM2_ESM.pdf]

The Description of Additional Supplementary Files

Supplementary Movie 1: Fog collection process of Cu-SHB-SLP film observed by ESEM.

Supplementary Movie 2: Fog collection process of Cu-SHBL-SLP film observed by ESEM.

Supplementary Movie 3: Actual images of the fog collection process on the 2D patterned Cu-SHBL-SLP film.

Supplementary Movie 4: Actual images of the fog collection process on the 2D patterned Cu-SHBL-SLP film with the θ=30°.
