# Supplementary material for: Preserving exposed hydrophilic bumps on multi-bioinspired slippery surface arrays unlocks high-efficiency fog collection and photocatalytic cleaning
Source: Nat Commun. 2025 Nov 6;16:9793. doi: 10.1038/s41467-025-65169-1 (PMC12592700; doi:10.1038/s41467-025-65169-1)
Supplement: Supplementary file 7 — Reporting Summary [file 41467_2025_65169_MOESM7_ESM.pdf]

nature portfolio

Corresponding author(s): Yan Yan; Jiangdong Dai

Last updated by author(s): Sep 1, 2025

Reporting Summary

Nature Portfolio wishes to improve the reproducibility of the work that we publish. This form provides structure for consistency and transparency in reporting. For further information on Nature Portfolio policies, see our Editorial Policies and the Editorial Policy Checklist.

Statistics

For all statistical analyses, confirm that the following items are present in the figure legend, table legend, main text, or Methods section.

n/a	Confirmed	
☒	☐	The exact sample size (n) for each experimental group/condition, given as a discrete number and unit of measurement
☒	☐	A statement on whether measurements were taken from distinct samples or whether the same sample was measured repeatedly
☒	☐	The statistical test(s) used AND whether they are one- or two-sided<br>*Only common tests should be described solely by name; describe more complex techniques in the Methods section.*
☒	☐	A description of all covariates tested
☒	☐	A description of any assumptions or corrections, such as tests of normality and adjustment for multiple comparisons
☒	☐	A full description of the statistical parameters including central tendency (e.g. means) or other basic estimates (e.g. regression coefficient) AND variation (e.g. standard deviation) or associated estimates of uncertainty (e.g. confidence intervals)
☒	☐	For null hypothesis testing, the test statistic (e.g. F, t, r) with confidence intervals, effect sizes, degrees of freedom and P value noted<br>*Give P values as exact values whenever suitable.*
☒	☐	For Bayesian analysis, information on the choice of priors and Markov chain Monte Carlo settings
☒	☐	For hierarchical and complex designs, identification of the appropriate level for tests and full reporting of outcomes
☒	☐	Estimates of effect sizes (e.g. Cohen's d, Pearson's r), indicating how they were calculated

Our web collection on statistics for biologists contains articles on many of the points above.

Software and code

Policy information about availability of computer code

Data collection	No data collection software was used
Data analysis	Data analysis and visualization were performed using Origin 2018 and ImageJ

For manuscripts utilizing custom algorithms or software that are central to the research but not yet described in published literature, software must be made available to editors and reviewers. We strongly encourage code deposition in a community repository (e.g. GitHub). See the Nature Portfolio guidelines for submitting code & software for further information.

Data

Policy information about availability of data

All manuscripts must include a data availability statement. This statement should provide the following information, where applicable:

- Accession codes, unique identifiers, or web links for publicly available datasets
- A description of any restrictions on data availability
- For clinical datasets or third party data, please ensure that the statement adheres to our policy

Data Availability Statement
The raw data supporting the findings of this study have been submitted alongside the manuscript. The data generated in this study are provided in the Source Data file.

Research involving human participants, their data, or biological material

Policy information about studies with human participants or human data. See also policy information about sex, gender (identity/presentation), and sexual orientation and race, ethnicity and racism.

Reporting on sex and gender	Our research does not involve sex and gender
Reporting on race, ethnicity, or other socially relevant groupings	Our research does not involve race, ethnicity, or other socially relevant groupings
Population characteristics	Our research does not involve population characteristics
Recruitment	Our research does not involve recruitment
Ethics oversight	Our research does not involve ethics oversight

Note that full information on the approval of the study protocol must also be provided in the manuscript.

Field-specific reporting

Please select the one below that is the best fit for your research. If you are not sure, read the appropriate sections before making your selection.

☐ Life sciences ☐ Behavioural & social sciences ☒ Ecological, evolutionary & environmental sciences

For a reference copy of the document with all sections, see nature.com/documents/nr-reporting-summary-flat.pdf

Ecological, evolutionary & environmental sciences study design

All studies must disclose on these points even when the disclosure is negative.

Study description	Our research is to study the fog collection performance of the prepared membranes. Each experiment was measured at least three times.
Research sample	The prepared fog collection membrane was fabricated in the same conditions to ensure the uniform sample.
Sampling strategy	Achieved by weighing the collected fog water.
Data collection	The experimental data were recorded by the first author (Junda Wu) during the fog collection process by pen and paper, and each sample was tested at least three times, as can be seen from the error bars in the manuscript figures.
Timing and spatial scale	All laboratory experiments and performance tests on the fabricated samples were conducted between March 2023 and September 2024, mainly in the laboratory environment. For each sample, testing was conducted over three independent 24-hour periods to ensure reproducibility. The mass of collected water was recorded at 10-minute intervals using a high-precision electronic balance. The experiments were conducted within a sealed environmental chamber with controlled conditions (temperature: 25 ± 1 °C; relative humidity: 90 ± 2%).
Data exclusions	No data exclusions.
Reproducibility	The fog harvesting experiments for each type of bio-inspired surface were independently repeated three times (n = 3). Each independent replicate involved the fabrication of a new sample from scratch and its subsequent testing in the environmental chamber. This approach ensures that our results account for the inherent variability in the material fabrication process.
Randomization	Following the normal randomness distribution of the collection experiment.
Blinding	It was not possible to blind the investigators during data collection because the experimental groups had gross morphological differences (various samples) that were impossible to conceal.

Did the study involve field work? ☐ Yes ☒ No

Reporting for specific materials, systems and methods

We require information from authors about some types of materials, experimental systems and methods used in many studies. Here, indicate whether each material, system or method listed is relevant to your study. If you are not sure if a list item applies to your research, read the appropriate section before selecting a response.

Materials & experimental systems

n/a	Involved in the study
☒	☐ Antibodies
☒	☐ Eukaryotic cell lines
☒	☐ Palaeontology and archaeology
☒	☐ Animals and other organisms
☒	☐ Clinical data
☒	☐ Dual use research of concern
☒	☐ Plants

Methods

n/a	Involved in the study
☒	☐ ChIP-seq
☒	☐ Flow cytometry
☒	☐ MRI-based neuroimaging

Plants

Seed stocks	Our research does not involve seed stocks
Novel plant genotypes	Our research does not involve novel plant genotypes
Authentication	Our research does not involve authentication
